# Case Report: Trastuzumab Treatment in Adenosquamous Carcinoma of the Extrahepatic Biliary Tract With Her-2 Amplification

**DOI:** 10.3389/fonc.2021.538328

**Published:** 2021-02-25

**Authors:** Ye Hong, Xiaofen Li, Dan Cao

**Affiliations:** Department of Medical Oncology, Cancer Center, West China Hospital, Sichuan University, Chengdu, China

**Keywords:** extrahepatic cholangiocarcinoma, trastuzumab, PFS, Her-2 amplification, adenosquamous carcinoma

## Abstract

Extrahepatic cholangiocarcinoma (ECC) is an uncommon and devastating malignancy that mainly consists of adenocarcinoma. Adenosquamous carcinoma is a rare histologic type and accounts for 2–5% of ECC. It reports that 3.6–8.5% of ECC patients carry Her-2 amplification. A 45-year-old woman was admitted to our hospital because of jaundice. Abdominal computerized tomography (CT) suggested extrahepatic biliary tract mass. The patient received surgery and pathological examination confirmed adenosquamous carcinoma. Fluorescence in Situ Hybridization (FISH) and Next-generation sequencing showed the tumor had Her-2 amplification. One month after the operation, CT demonstrated distant lymph nodes metastases (cT3N1M1, stage IV). The patient received gemcitabine and cisplatin combined with targeted therapy of trastuzumab. After three cycles of treatment, the evaluation of response was stable disease (SD). The progression-free survival of 1st line treatment (PFS1) reached 5 months with five cycles of treatment. After progression, the patient received three cycles of albumin-bound paclitaxel combined with S-1 and trastuzumab and concurrent chemoradiotherapy (S-1) because of serious backache. Now, the disease is stable, and the PFS of 2nd line treatment (PFS2) has reached 7 months.

## Introduction

Cholangiocarcinoma (CCA) is a rare cancer that is formed of intrahepatic cholangiocarcinoma (ICC) and extrahepatic cholangiocarcinoma (ECC) (1) with a poor prognosis (2). ECC is the most common type of cholangiocarcinoma. In the past several decades, the incidence and mortality rate of CCA had increased in the world, especially in North America and Asia (3). Adenocarcinoma is the most common histologic type and accounts for more than 90% of ECC, while adenosquamous carcinoma is an extremely rare type that accounts for 2–5% of ECC (4). Adenosquamous carcinoma consists of two kinds of malignant components—adenocarcinoma and squamous carcinoma—and it seems to have greater malignant potential than adenocarcinoma (5). There is no standard management for adenosquamous carcinoma due to the low incidence rate. The management of adenosquamous carcinoma always refers to the treatment of adenocarcinoma, which has no satisfactory effect and worsening survival than adenocarcinoma (6). Surgery is the only curative therapy, but most patients lose the chance of the operation at the time of diagnosing and even if patients receive surgery, they would quickly relapse (7). Systematic chemotherapy is the standard first-line treatment for unresectable and advanced CCA with poor response and survival (8, 9) and there is no standard second-line treatment (10). Thus, we urgently need to explore an effective treatment for adenosquamous carcinoma.

It has been reported that 3.6–8.5% of ECC patients carry Her-2 amplification (11, 12). Her-2 amplification is closely linked to the proliferation, motility, and invasiveness of cholangiocarcinoma (13) and targeting Her-2 can intensify the anti-tumor effect of chemotherapy (14). A serial of cases has reported the efficacy of trastuzumab for gallbladder carcinoma (15) (16). There is only one case that has reported the antitumor efficacy of trastuzumab combined with pertuzumab for an ECC patient (17). A phase 2 clinical trial concerning trastuzumab in combination with chemotherapy for biliary tract cancer is ongoing (18). This evidence all suggested targeting Her-2 may be an effective treatment for cholangiocarcinoma.

Herein we report the process of diagnosis and treatment of an adenosquamous carcinoma of the extrahepatic biliary tract with Her-2 amplification in our hospital

## Case Report

A 45-year-old woman searched for physician assistance in September 2018, presenting with back pain, vomiting, and nausea. Upper abdominal magnetic resonance imaging (MRI) showed acute pancreatitis, dilation of the pancreatic duct, intrahepatic bile duct, middle, and upper common bile duct. After symptomatic treatment and bile duct drainage were implemented in the local hospital, the patient was transferred to our hospital (West China Hospital, Sichuan University) for further diagnosis and treatment. Physical examination showed evident jaundice. Liver function test showed remarkably increased bilirubin, with total bilirubin 47.2 umol/L and direct bilirubin 46 umol/L. Cancer antigen19-9 (CA19-9) was 263.9 U/ml (normal range was < 22 U/ml). Chest computerized tomography (CT) suggested enlargement of lymph nodes in the left neck root and left axillary. Abdominal CT demonstrated a 2.7cm x1.3 cm, low density, vague boundary nodule in the pancreatic head and inferior common bile duct with thickened inferior common bile duct and dilated pancreatic duct. Multiple lymph nodes located at the posterior and inferior pancreas, para-abdominal aorta, and adjacent mesentery were obviously enlarged. After routine preoperative examination, pancreatic-duodenectomy plus hilar choledochoplasty, portal vein repair, and greater omentum lesion resection were performed on 31 Oct 2018. During the operation, a hard mass of about 3cmx2cm was palpated in the head of the pancreas and the lower segment of the common bile duct without obvious vascular invasion, the common bile duct was dilated with a diameter of about 2.5cm, and the wall of the common bile duct was thickened. Intraoperative frozen biopsy suggested adenosquamous carcinoma and incisal margins of the biliary and pancreatic duct were cancer-free. Postoperative pathological diagnosis confirmed moderate to poorly differentiated adenosquamous carcinoma of the bile duct, infiltrating the whole layer of the common bile duct and invading the ampulla, pancreatic parenchyma, and the fibrous connective tissue around the pancreas, with immunohistochemical (IHC) staining of CK7(+), CK19(+), CK20(partly +), P63(partly +), P40(partly +), CK5/6(partly +), DPC4(partly -), CDX2 (-), Her-2(squamous carcinoma was accounted for about 80%; adenocarcinoma was moderate to strong and accounted for about 30%, moderate to strong expression). No carcinoma was found in the stump of the greater curvature of the stomach, the lesser curvature of the stomach, the duodenum, and greater omentum lesion. Metastasis was found in one regional lymph node. Fluorescence in Situ Hybridization (FISH) and Next-generation sequencing (NGS) verified the amplification of Her-2 gene. Finally, the patient was diagnosed as Her-2 amplification adenosquamous of the extrahepatic biliary tract with regional lymph node metastasis.

**Figure 1:**
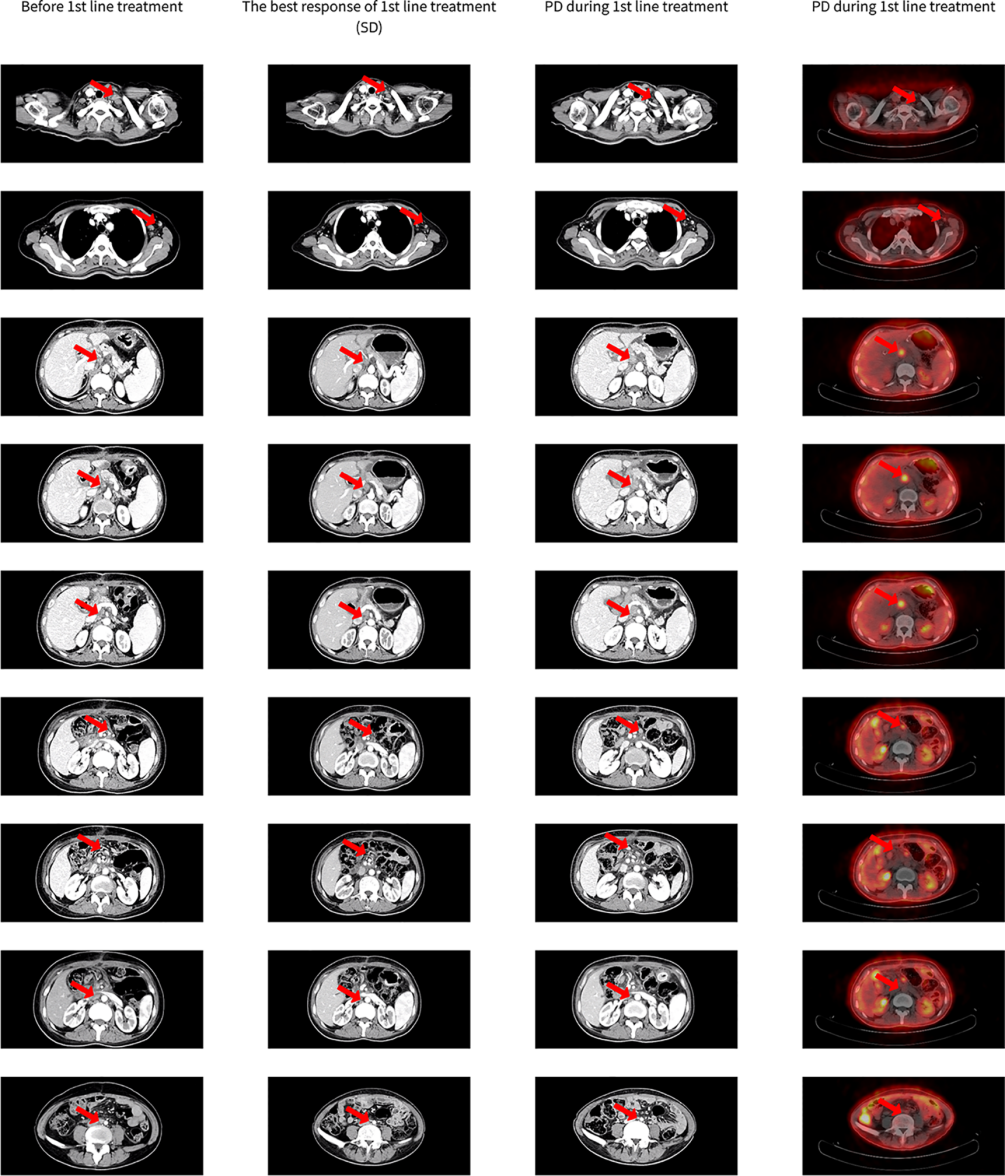
The response of 1st line treatment.

One month after the operation, chest and abdominal CT showed enlargement of multiple lymph nodes located in the left neck root, left axillary, and mesenteric and para-abdominal aortic (**Figure 1**). The patient was diagnosed as adenosquamous carcinoma of the extrahepatic biliary tract with multiple lymph nodes metastases (retroperitoneal, para-iliac, left neck root, and left axillary lymph nodes) harboring Her-2 amplification (cT3N1M1, stage IV). After a general discussion the patient received systemic chemotherapy consisting of gemcitabine 1000mg/m^2^ d1, d8 plus cisplatin 75mg/m^2^ combined with trastuzumab 4mg/kg d1 (6mg/kg first time), repeated every 3 weeks, the lymph nodes shrank evidently after three cycles of treatment and the best efficacy evaluation was stable disease (SD) according to RECIST 1.1 (**Figure 1**). But after five cycles of treatment, abdominal CT and PET-CT showed enlargement of para-abdominal aortic lymph nodes, and disease was evaluated progressed (**Figure 1**). The progression-free survival of first-line treatment (PFS1) was 5 months.

**Figure 2:**
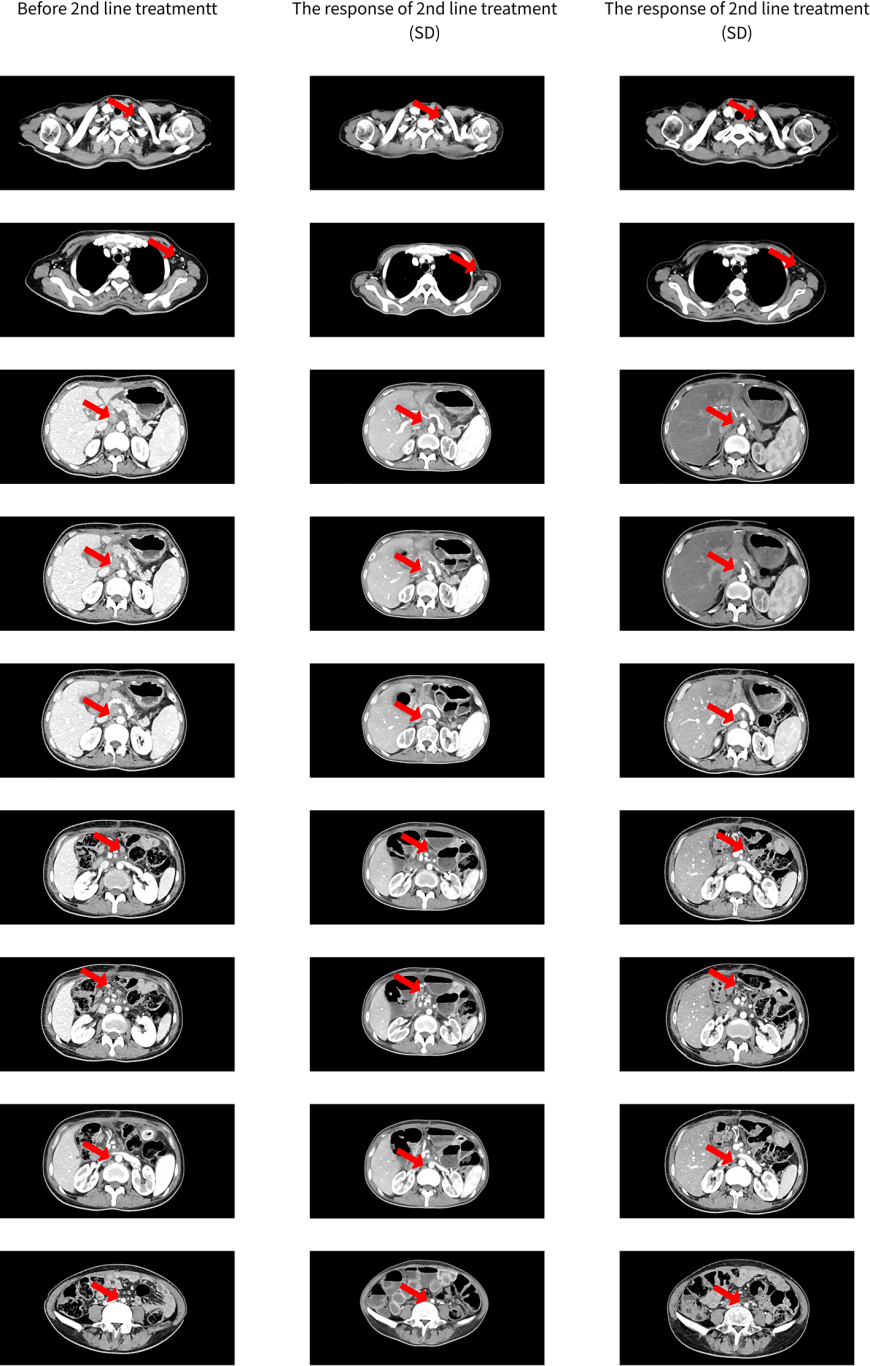
The response of 2nd line treatment.

After discussion with the patient and her family, second-line chemotherapy consisting with albumin-bound paclitaxel 125mg/m2 d1, d8 plus S-1 50mg twice a day d1-d14 and trastuzumab 4mg/kg d1, repeated every 3 weeks. To relieve severe backache, after receiving one cycle of systemic chemotherapy, radiotherapy (2Gy/30f/60Gy) of para-abdominal aortic lymph nodes with concurrent S-1 (50mg bid) were prescribed from June 21, 2019 to August 13, 2019. After finished radiotherapy, the evaluation of efficacy was stable disease (lymph node was smaller than before, **Figure 2**). Then the patient received continuation of albumin-bound paclitaxel plus S-1 and trastuzumab. The recent response evaluation was SD (**Figure 2**). The PFS2 is 7 months up to now.

## Discussion

Searching for electrical databases, we did not find a report of Her-2 amplification adenosquamous carcinoma of the extrahepatic biliary tract. As far as we know, this is the first report about Her-2 amplification adenosquamous carcinoma of the extrahepatic biliary tract. Our patient received two lines of chemotherapy combined with targeted therapy of trastuzumab and radiotherapy. Now the disease is stable and the patient has lived for about 14 months. Besides, the PFS1 was 5 months and the PFS2 is 7 months up to now.

At present, gemcitabine combined with cisplatin is the standardized 1st line treatment for advanced ECC. However, the prognosis is very poor (median overall survival was 11.7 months and median progression-free survival was 8 months) and there is no standard 2nd line treatment. Adenosquamous carcinoma has no standard treatment because of the low incidence rate, which refers to the treatment of adenocarcinoma. It was reported that the median overall survival of advanced adenosquamous carcinoma of the extrahepatic biliary tract was 6–7 months (19, 20) and the 5-year survival rates of adenocarcinoma and adenosquamous carcinoma were 23–44% and 16%, respectively, which suggested adenosquamous carcinoma has a worsening prognosis than adenocarcinoma.

Her-2 is a member of the epidermal growth factor receptor family. It was reported that the rates of Her-2 amplification ranged from 0.5% to 69% in different solid tumors (21). Her-2 is an efficacy-predictor of trastuzumab for certain solid tumors, such as breast cancer and gastric cancer. A series of cases of Her-2 overexpressed gallbladder carcinoma have reported satisfactory efficacy of trastuzumab and basic research has found trastuzumab could intensify the efficacy of gemcitabine. This evidence all suggest that trastuzumab is a potentially effective treatment for Her-2 amplification cholangiocarcinoma. What we should pay attention to is that the expression of Her-2 in squamous cell carcinoma and adenocarcinoma is different. It has been reported that Her-2 amplification was more frequent in adenocarcinoma in various solid tumors, which implied the heterogeneity of cancer (22). Our patient is an adenosquamous carcinoma that has a different level of Her-2 amplification between adenocarcinoma and squamous carcinoma components (squamous carcinoma was moderate to strong and accounted for about 80%; adenocarcinoma was moderate to strong and accounted for about 30%), which may influence the efficacy of trastuzumab.

Our patient received the gemcitabine plus cisplatin and trastuzumab as the 1st line treatment and the PFS was 5 months, which was less than the results (PFS 8 months) of the ABC-02 trial, but the majority of the enrolled patients of the trial had adenocarcinoma, which has a better prognosis. And after progression, the patient received chemotherapy (albumin-bound paclitaxel combined with S-1) and targeted therapy (trastuzumab) as the 2nd line treatment and radiotherapy for regional lymph nodes and now the disease is still stable and the PFS has reached 7 months. Until now there is no standard second-line treatment for cholangiocarcinoma. It was reported that the median PFS of cholangiocarcinoma patients who received different kinds of second-line treatments were 3.2 months (95% CI 2.7–3.7 months) which were significantly shorter than 7 months of our study. These results suggested our patient acquired an effective treatment. None of the lymph node lesions progressed during second-line treatment, indicating the effectiveness of systematic treatment.

In conclusion, adenosquamous carcinoma of the biliary tract with Her-2 overexpression is a rare disease with a poor prognosis and no standard treatment. Trastuzumab combined with chemotherapy and radiotherapy was probably effective for Her-2 amplification adenosquamous carcinoma of the extrahepatic biliary tract.

## Ethics Statement

The study involving human participants was reviewed and approved by West China Hospital of Sichuan University Biomedical Research Ethics Committee. The participant provided her written informed consent to participate in this study. Written informed consent was obtained from the individual(s) for the publication of any potentially identifiable images or data included in this article.

## Author Contributions

DC performed the radiological analysis of MRI CT and PET-CT images. YH and XFL wrote the first draft of the manuscript. DC wrote sections of the manuscript. All authors contributed to the article and approved the submitted version.

## Conflict of Interest

The authors declare that the research was conducted in the absence of any commercial or financial relationships that could be construed as a potential conflict of interest.
